# App-Based Mindfulness Training Predicts Reductions in Smoking Behavior by Engaging Reinforcement Learning Mechanisms: A Preliminary Naturalistic Single-Arm Study

**DOI:** 10.3390/s22145131

**Published:** 2022-07-08

**Authors:** Veronique A. Taylor, Ryan Smith, Judson A. Brewer

**Affiliations:** 1 Mindfulness Center, School of Public Health, Brown University, 121 South Main Street, Providence, RI 02903, USA; judson_brewer@brown.edu; 2 Laureate Institute for Brain Research, 6655 South Yale Ave., Tulsa, OK 74136, USA; rsmith@laureateinstitute.org; 3 Warren Alpert Medical School, Brown University, 222 Richmond Street, Providence, RI 02903, USA

**Keywords:** digital therapeutics, tobacco smoking, mindfulness training, reinforcement learning, computational modeling, reward value

## Abstract

Mindfulness training (MT) has been shown to influence smoking behavior, yet the involvement of reinforcement learning processes as underlying mechanisms remains unclear. This naturalistic, single-arm study aimed to examine slope trajectories of smoking behavior across uses of our app-based MT craving tool for smoking cessation, and whether this relationship would be mediated by the attenuating impact of MT on expected reward values of smoking. Our craving tool embedded in our MT app-based smoking cessation program was used by 108 participants upon the experience of cigarette cravings in real-world contexts. Each use of the tool involved mindful awareness to the experience of cigarette craving, a decision as to whether the participant wanted to smoke or ride out their craving with a mindfulness exercise, and paying mindful attention to the choice behavior and its outcome (contentment levels felt from engaging in the behavior). Expected reward values were computed using contentment levels experienced from the choice behavior as the reward signal in a Rescorla–Wagner reinforcement learning model. Multi-level mediation analysis revealed a significant decreasing trajectory of smoking frequency across MT craving tool uses and that this relationship was mediated by the negative relationship between MT and expected reward values (all *p*s < 0.001). After controlling for the mediator, the predictive relationship between MT and smoking was no longer significant (*p* < 0.001 before and *p* = 0.357 after controlling for the mediator). Results indicate that the use of our app-based MT craving tool is associated with negative slope trajectories of smoking behavior across uses, mediated by reward learning mechanisms. This single-arm naturalistic study provides preliminary support for further RCT studies examining the involvement of reward learning mechanisms underlying app-based mindfulness training for smoking cessation.

## 1. Introduction

Tobacco use is a major cause of preventable death and disease in the US, accounting for over 8 million deaths per year across the globe [1]. Although the majority (approximately 70%) of tobacco smokers report the desire to quit [2] less than 5% of quitting attempts (without support resources) are successful [1]. Therefore, it is critical to conduct basic and translational research that can empirically guide the development and optimization of behavioral interventions for smoking cessation.

Current behavioral treatments for smoking cessation (e.g., cognitive–behavioral or behavior modification therapy) involve, for example, cognitive strategies to change beliefs about the benefits of smoking, or avoidance strategies to divert attention from craving-triggering cues [3,4]. However, these have yielded modest success rates and relatively low abstinence rates for cognitive-based behavioral therapies and psychosocial approaches [4,5,6,7]. One reason for this limited success may be that these tools do not target core reinforcement learning (RL) mechanisms in addiction—such as associative learning and reward valuation processes—which contribute to the formation of tobacco smoking habits and their maintenance [8].

Indeed, from a RL perspective, the formation of smoking as a habit partly stems from associating smoking behaviors with either negative affect/experiences (during a stressful workday) or positive affect/experiences (feeling relaxed at a party with friends) [9]. As a result, the person learns that smoking “makes them feel better”, either by generating or maintaining positive affect (positive reinforcement), or reducing negative affect (negative reinforcement), and creates an associative memory between the behavior (smoking) and this short-term outcome (“feeling better”). Thus, specific cues repeatedly present when smoking become predictors of smoking behavior and gain value either by generating positive affect or reducing negative affect [10,11]. In turn, cues eliciting these affective states (e.g., getting yelled at by a boss) can trigger cigarette cravings, leading the person to smoke, and the habitual behavior becomes further strengthened through positive or negative reinforcement. Through repetition, this reinforcement learning process becomes automatic and performed outside conscious awareness (for review of associative learning “addictive loop” for nicotine dependence, see [8]).

Consequently, future exposure to cues (either internal, such as affective states, or external stimuli, such as viewing “cool” kids at school smoking) become more likely to induce craving and subsequent smoking behaviors [10]. Importantly, when sufficiently reinforced, smoking habits tend to persist despite being devalued. That is, reward value may be high in initial stages of habit acquisition due to positive/hedonic state induction or relief from negative states; yet, over time, these reinforced habits can persist despite carrying less reward value than when initially formed—a process defined as reward devaluation [12]. Thus, smoking may persist even though it has become less rewarding, or despite its negative consequences. More specifically, the behavior of smoking may persist out of habit or automaticity, but the person may no longer like smoking or the consequences resulting from the behavior.

Mindfulness-based interventions are emerging as approaches for smoking cessation (for reviews, see [13,14,15]). While Maglione and colleagues’ literature review did not find significant effects of mindfulness on tobacco use with respect to comparators [15], other systematic reviews on randomized controlled trials examining the impact of 8 weeks of mindfulness-based stress reduction programs have determined significant improvements in craving, relapse rate [13] and abstinence rates assessed at long-term follow-up [14]. One particular study found reductions in smoking from mindfulness training in college students over a brief duration of time (7 days [16]). The involvement of reward-related processes in the application of mindfulness for addiction has also been described, as well as its role in “restructuring reward” and a specific mindfulness-based intervention (Mindfulness-Oriented Recovery Enhancement, “MORE”) helping individuals shift from synthetic or drug-induced rewards to natural rewards (e.g., a beautiful landscape) [17]. Significant reductions in tobacco use in participants having undergone an 8-week MORE compared to a control group have been previously reported [18].

According to a similar reward-based framework, it has also been described that mindfulness may be beneficial in countering habit-driven behaviors by specifically targeting reinforcement learning systems—and more specifically by recalibrating a behavior’s value by directing attention to how rewarding that behavior actually is [8,19]. This specific focus on the outcome of the behavior would, in turn, generate a negative prediction error [20,21]—in other words, if the person does not feel as content after performing their behavior as they expected to, this would induce a steep decrease in reward value until the next encounter with the behavior.

This mechanism of action has been previously described and supported in helping individuals who struggle with frequent cravings for unhealthy foods [22]. This study showed a decreasing slope trajectory of the reward value of eating across uses of a mindful eating app-based craving tool, which predicted corresponding decreases in maladaptive eating behavior [22]. Mindfulness may convey this benefit precisely because it involves the voluntary deployment of awareness on present-centered experiences in a non-judgmental and accepting manner [23], instead of focusing on will-power to overcome cravings. As opposed to the active avoidance of cues or changes in affect, this approach fosters acceptance of craving—the experience of being with craving or affective states—rather than reacting to them by smoking [8,19]. Supporting the mindfulness approach to behavior change, previous work has shown that participants assigned to the mindfulness training group exhibited 31% point prevalence abstinence rate from a 4-week intervention assessed at 17 weeks follow-up compared to 6% in a group assigned to current standard smoking cessation treatment (Freedom from smoking intervention by the American Lung Association [24]).

App-delivered mindfulness training (MT) offers the advantage of facilitating mindful attention to smoking cues, experiences of craving, and the affective consequences of smoking behavior as they occur in real time and in their ecological, real-world context. This modality also offers the advantage of being able to precisely track the dose/frequency of treatment delivery and to ensure its content standardization across participants. To this end, the “Craving to Quit” (C2Q) program [25] was designed as a smoking cessation tool to foster the development and practice of mindful awareness of experiences of cravings immediately as they arise. The program involves two components: helping individuals (1) recognize cues that trigger cravings and responses to cravings, and (2) recognize the outcome resulting from the behavior (Do I really like doing this?). Critically, this second component involves guiding participants to focus on the full range of (affective) outcomes of the behavior by becoming aware of their level of contentment after choosing to smoke, rather than focusing cognitively or intellectually on the negative health effects of smoking.

For instance, a person may experience temporary relief from unpleasant withdrawal symptoms by smoking, but at the same time find that mindful smoking leaves unpleasant smells, tastes and sensations in their mouth, throat, and lungs. In doing so, the reward value—that is, the expected amount of reward to be obtained from performing the behavior—would become recalibrated to a lower value based on a negative prediction error and diminish the probability of the behavior’s occurrence in the future. This is consistent with a description of mindful smoking offered by one example patient: “Mindful smoking smells like stinky cheese and tastes like chemicals. YUCK.”, p. 201 [19]. Indeed, by paying attention to the experience of smoking and its consequences, the reward value may be recalibrated and attenuate any reinforcing effects it may have otherwise had. As a result, the habit maintenance cycle can be disrupted. In other words, the next time a smoking cue is encountered, a weakened representation of the reward value for smoking may become activated, decreasing the likelihood of reacting by smoking. However, mechanisms relating MT to reinforcement learning have not been studied as they relate to smoking cessation.

Here, we used computational models of reinforcement learning (Rescorla–Wagner) to determine changes in reward value for smoking within individuals using the C2Q program’s craving tool. This well-established learning model [26] suggests that learning occurs at a steeper rate when an outcome is most surprising. In other words, the reward value is updated most steeply in trials in which there is a large discrepancy (prediction error) between the expected reward value and the actual level of reward encountered from the experience (reflected in contentment levels from smoking).

A distinction that is important to make is that between expected reward values, updated as a function of the last experience with the behavior’s outcome (level of contentment felt), and present-moment reward values reflected in the urge or “wanting” aspect to engage in the behavior at the next encounter with craving-inducing cues. The latter construct can be reflected in craving levels just prior to engaging with the behavior, which would reflect the updated expected level of reward from the last encounter with the behavior (as a function of contentment levels), in conjunction with present-moment factors that could influence motivational states (e.g., stress levels, withdrawal symptoms). For instance, a person may have a relatively low reward value for smoking from having experienced discontentment with smoking at a given use of the craving tool, yet on the next encounter with a craving-inducing cue, a higher level of reward values may be activated due to other motivational factors that might induce variations in urges to smoke (e.g., physiological withdrawal, stress, and physiological stress markers [27,28,29]). Thus, both expected reward values and present-moment reward values can be assessed to determine the correspondence between these two constructs and if they were influenced in the same way by the use of the mindful smoking craving tool.

In the present study, data were acquired from participants in the general population (n = 108) as they obtained and used the C2Q program on their own initiative in order to obtain a naturalistic depiction of the program’s effect on reward value and behavior. Computational modeling was used to estimate expected reward values and how they changed as a result of self-reported contentment levels after each instance of choosing to smoke. We hypothesized that decisions to smoke while using the app would decrease across time, and that this decreasing slope as a function of the number of mindful craving tool uses would be mediated by reductions in expected reward values (i.e., that the learning model would account for behavior change). Finally, we explored the correspondence between expected reward values and present-moment reward values across uses of the mindful smoking craving tool.

## 2. Materials and Methods

### 2.1. Participants

This study’s inclusion criteria were defined broadly as participants having initiated the start of the Craving To Quit (Sharecare, Inc., Atlanta, Georgia, USA) app between 31 May 2019 and 6 July 2020. To obtain sufficient data depicting the time course of learning and behavioral trajectories, we included subjects who had used the tool a minimum number of 10 times. Two participants with a deviant (>3 SD) number of trials were excluded, leaving a total of 108 participants for data analysis. The analyses reported here were also conducted with the inclusion of outlier participants, after which results remained essentially unchanged. These outlier participants were therefore excluded to obtain a more homogeneous range of trial numbers and app usage experience across the sample. On average, participants (sex: 57 females/48 males, 3 identified as “other”) were 45.57 years of age (SD = 11.65) and used the craving tool 26.74 times (SD = 21.70).

Participants obtained C2Q on their own initiative, and their behavior was naturalistically observed as they used the app’s craving tool upon the experience of a craving for cigarette smoking.

### 2.2. Craving to Quit (C2Q) Program: Mobile Mindfulness Training with Experience Sampling

The Craving to Quit smartphone app is a program designed to help people become aware of their habits, how habits are formed/maintained through reinforcement learning, and how mindful awareness of habits and behaviors can be applied to override cravings and urges to smoke. At the start of the program, a brief video tutorial explains the characteristics of the app to ensure the user is ready to begin the program. The app offers mindfulness training modules in the form of brief video capsules created to teach mindfulness for smoking cessation skills. The C2Q program involves 22 training modules (5–15 min each) consisting of audio and video content as well as animations and exercises designed to educate people on how and why they smoke (the cues and triggers that induce their cravings/behavior, and automatic ways in which they smoke) and to develop skills to practice mindful awareness. Participants are only allowed to access one new module per day but can view previously viewed modules as often as desired. The general aim of the app is to educate people about the concept and practice of mindfulness and applying mindful awareness to present-moment experiences, as well as teaching various standard types of meditation practices. A specific focus is placed on directing mindful awareness towards smoking habits and behaviors. Additional features of the app include access to a social online community platform, as well as others such as goal reminders or check-ins which can be used or adjusted on an individual basis (see [25] for an in-depth description of the app’s and modules’ contents).

**Figure 1:**
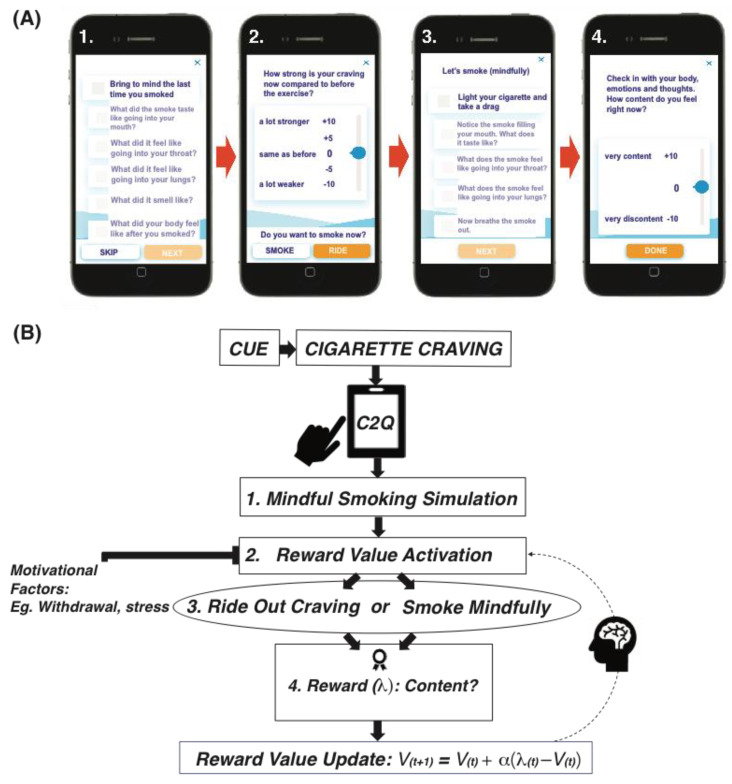
Prototypical craving tool use (**A**) and theoretical model (**B**) depicting the sequence of events/processes involved during the craving tool exercise. C2Q: Craving to Quit mindfulness training program. Expected reward values are denoted as “V” in the equation depicting the Rescorla-Wagner reinforcement learning model used to compute values for each action (smoking or not smoking by riding-out their cravings with the brief informal mindfulness RAIN exercise) at each craving tool use (“t”). Expected values are computed as a function of the prediction error, which is defined as the discrepancy between the outcome (“λ“: contentment levels after the behavior) and the expected value obtained from the previous craving tool use. The prediction error term is weighed by an individually fixed learning rate parameter (“α “).

The program’s craving tool involves a mindful smoking exercise designed to help people become more fully aware of the phenomenological qualities experienced during and after smoking. The tool’s configuration is similar to that of the mindful eating craving tool embedded in the Eat Right Now app-based mindfulness training program, which we previously examined with respect to the reward value assigned to eating behaviors [22]. Figure 1 shows a prototypical craving tool use and theoretical model of processes/events involved in each step. The tool is designed to be used when participants experience cigarette cravings “in-vivo”, which first invites them to click “Want-O-Meter” on the app’s dashboard. The tool then invites participants to go through a mindful smoking simulation by bringing to mind the last time they had smoked, in order to bring into consciousness or activate the reward value representation of smoking stored with the last experience from the behavior (Figure 1A. Screenshot1). They are then invited to pay attention to this last experience and its outcome. More precisely, they are invited to bring to mind the sensations encountered while smoking (e.g., how it had felt in their body (mouth, throat and lungs) and the way it smelled). It also asks them to bring to mind the experience (body sensations, thoughts, emotions) encountered after smoking.

Following the mindful smoking simulation exercise, participants are instructed to indicate their level of craving (in comparison with their baseline craving intensity prior to the initiation of the exercise) (Figure 1A. Screenshot2). This urge/wanting aspect to engage in smoking serves as a read-out of in-the-moment reward value for smoking. Following this, participants are given the option to either smoke mindfully, or “ride out their craving” with a brief informal mindfulness practice (Figure 1A. Screenshot2). If they opt to smoke mindfully, they are asked to pay attention to the qualities experienced during smoking (e.g., sensations, smells, perceptions. Figure 1A, Screenshot3), following which they are asked to check in with their body, thoughts and emotions and provide a rating of how content they feel after smoking (Figure 1A. Screenshot4).

If they opt not to smoke, they are directed to complete a brief informal mindfulness practice (RAIN exercise) designed to “ride-out” cravings. The RAIN exercise is taught within the program’s 4th training module and is a brief audio-guided mindfulness exercise designed to help participants Recognize, Accept, Investigate, and Note the phenomenological qualities of their craving (e.g., bodily sensations) as they are experienced in the present moment. Following the RAIN exercise, they are asked to check in with their body, thoughts, and emotions, and provide a rating of how content they feel in the present moment. Finally, an option is given to either resume the exercise, or to check in on their present-moment experience and rate contentment levels 5 and 15 min later.

### 2.3. Measures

#### 2.3.1. Demographic and Intervention Completion Variables

Age and sex were recorded via the app’s electronic database, as well as the number of mindful training modules completed (M = 18.93, SD = 8.09, range: 3–28). The next set of measures listed are experience sampling measures, which are as those of our mindful eating craving tool used and described in our previous article [22].

#### 2.3.2. Present-Moment Reward Values

After bringing to mind the last experience with smoking (i.e., to activate the behavior’s reward value) (Figure 1A. Screenshot1), participants rated their level of craving anchored in baseline level of craving (before the initiation of the exercise). In order to do so, they entered their rating by answering the question “How strong is your craving now compared to before the exercise?”. A Likert scale ranging from −10 to +10 (−10 = *a lot weaker*, 0 = *same as before*, +10 = *a lot stronger*) was used to enter their rating (Figure 1A. Screenshot2). For data analysis, participant’s scores were rescaled between −1 and 1.

#### 2.3.3. Smoking Behavior

After paying mindful attention to the simulated experience of smoking, participants were prompted by the question “Do you want to smoke right now?” and decisions to smoke or not were recorded as 1 (“Smoke”) or 0 (“RIDE”) (Figure 1A. Screenshot2).

#### 2.3.4. Contentment from Smoking or Riding Out Cravings

After either having smoked mindfully or refrained from smoking, participants were asked to rate their levels of contentment. To this end, they were asked to pay mindful attention towards the present-moment phenomenological qualities (bodily sensations, thoughts and emotions), experienced after either smoking or riding out their craving with the RAIN exercise. Following this, they were asked to enter a rating indicating their level of contentment experienced from smoking (Figure 1A. Screenshot4). In order to do this, participants received the prompt “Check in with your body, thoughts and emotions. How content do you feel right now?”. Their contentment rating was then entered with a Likert Scale (−10: very discontent, +10: very content). For data analysis purposes, participants’ scores were rescaled between −1 and 1.

#### 2.3.5. Expected Reward Values from Smoking

Expected reward values for smoking were estimated using the computational learning models described in the following Data Analysis section.

### 2.4. Data Analysis

The data analyses performed and described here (computational reinforcement learning models and multi-level regression analyses) are similar to those performed in our previous article in which expected values of maladaptive eating behaviors were examined from participant’s use of a mindful eating craving tool [22].

#### 2.4.1. Learning Model Descriptions

As in our previous work, expected reward values for smoking were estimated using a Rescorla-Wagner model of reinforcement learning [26]. RL models were conducted using MATLAB Version 2020a (The MathWorks, Inc., Natick, MA, USA) and in-house scripts adapted from those used in [22]. At each craving tool use (modeled as a trial *t*), the expected value for each action (a) (smoking/riding out craving) was computed according to Equation (1).

(1) $$V_{a,\;t+1}=V_{a,\;t}+\;\alpha \;\left(\lambda_{t}-V_{a,t}\right)$$

Specifically, this model assumes that the expected amount of reward to be obtained on the next encounter with a behavior (denoted by $V_{a,\;t+1}$) is updated as a function of the reward value determined at the current experience with the behavior ($V_{a,\;t}$) as well as a prediction error term $\left(\lambda_{t}-V_{a,\;t}\right)$. This prediction error reflects the “surprisingness” of the behavior’s outcome, i.e., the discrepancy between the expected outcome from a smoking behavior at a given trial ($V_{a,\;t}$) and the actual outcome experienced from the behavior at that trial *t* ($\lambda_{t}$). The prediction error is then scaled by a learning rate parameter (α) that controls the magnitude of the update at each time point.

Here, $\lambda_{t}$ at a given craving tool use (*t*) reflects the level of contentment experienced after having either smoked or ridden out a craving and will induce a steep update if there is a high discrepancy between the observed level of contentment and the level of contentment that was expected ($V_{a,\;t}$). For instance, if high levels of reward are anticipated from smoking, but strong discontentment is experienced after *actually* smoking the craved cigarette, a steep decrease in smoking reward value would be observed because of the high discrepancy between the expected vs. actual outcome from the behavior.

At each craving tool use (trial), the likelihood of selecting the observed action given each action’s expected values was computed using the softmax function (Equation (2)).

(2) $$P({\mathrm{smoke}}_{t}\;|{\;V}_{smoke,t},{\;V}_{no\;smoke,t})=\exp\;\left(\beta \times {\;V}_{smoke,t}\right)/(\;\exp\;\left(\beta \times {\;V}_{smoke,t}\right)+\;\exp\;\left(\beta \times {\;V}_{no\;smoke,t}\;\right)$$

At each trial, the likelihood that a participant would select a given action (smoking vs. not smoking by riding out craving with the RAIN exercise) was modulated by an inverse temperature parameter (denoted by β), which controls the determinacy with which the highest-value action will be chosen.

For each participant, free parameters were estimated for the following variables: learning rate, initial expected smoking reward value for each action, and inverse temperature parameter. To estimate free parameters, an optimization search function was used that maximized the summed likelihood of observed actions for each participant.

We used an alternative type of reinforcement learning model (Rescorla–Wagner/Pearce–Hall Hybrid Model) to compare learning models’ goodness of fit to the data. This Rescorla–Wagner/Pearce–Hall Hybrid model (depicted in Equations (3) and (4)) is similar to the previously described Rescorla–Wagner model, but at each trial, it assumes that the learning rate is dynamically modulated by an associability term ($assoc_{a,t}$).

(3) $$V_{a,\;t+1}=V_{a,\;t}+assoc_{a,t}\times \;\alpha \;\left(\lambda_{t}-V_{a,t}\right)\;$$

(4) $$assoc_{a,t+1}=\gamma \left|\;\left(\lambda_{t}-V_{a,t}\right)\;\right|+\left(1\;–\;\gamma \right)\times assoc_{a,t}$$

The associability term is updated as a function of the prediction error that is experienced on the previous trial (*t* − 1). For example, in a trial in which high uncertainty levels are experienced (due to high prediction error) the associability term would increase during the next trial, and correspondingly increase the extent to which the prediction error would update the expected value. Associability has been interpreted as involving attention processes, such that highly surprising events attract attention and increase learning rate [30]. This is because highly surprising events can be taken to signal that there is a strong need to improve one’s model so as to adaptively guide behavior [30]. The associability term’s rate at which it is updated at each trial is further controlled by a fixed parameter (γ, bounded between 0 and 1).

The softmax rule (as described for the Rescorla–Wagner Model above) was used to compute the likelihood of selecting an action given the reward values assigned to each action. In the same way as for the Rescorla–Wagner, the same free parameters were estimated with the addition of the γ parameter.

#### 2.4.2. Model Comparison and Selection

Indices of model fits (Aikake information criterion, “AIC”) were computed for each subject and compared between models to determine the model that best fit the data. A paired sample *t*-test between AIC of the RW and Rescorla–Wagner/Pearce–Hall Hybrid Model indicated that the AIC was significantly lower (*p* < 0.001), and thus a better fit to the data, for the Rescorla–Wagner model compared to the Rescorla–Wagner/Pearce–Hall Hybrid Model. We therefore selected the Rescorla–Wagner model to estimate expected reward values to include in data analyses with craving tool measurements.

Averaged free parameter estimates across the sample for the selected model are listed as follows: α=0.13, β=16.68, $V_{no\;smoke,0}=0.23$, $V_{smoke,0}=0.41$. The average probability that the action predicted by the model was selected was 0.77.

To confirm that parameter estimates were reliable within the range of values and trial numbers in our study, we performed parameter recoverability analyses. Specifically, we used the model to generate simulated behavior and reward signals under a range of parameter combinations (i.e., α, v0_not_smoking, v0_smoking, β) and trial numbers observed in our participants. Parameter combinations and numbers of trials in these simulations were randomly selected from 50 participants in our sample (α values ranged from 0–1; v0_not_smoking values ranged from −1 to 1; v0_smoking ranged from −1 to 1; β ranged from 0.24–20; trial numbers ranged from 10 to 106). We then performed parameter estimation on this simulated behavior to confirm that the true (generative) parameters and estimated parameters were significantly correlated. Behavior at each simulated trial was generated based on sampling from the probability distribution over actions within the action model (Equation (2)). Reward signals were generated using random values between −1 and 0 for simulated smoking trials, and between 0 and +1 for simulated non-smoking trials. This was based on the expectation that smoking behaviors would generate lower reward signals when individuals used our mindful smoking craving tool, and that non-smoking behaviors would instead induce positive reward signals. These analyses revealed that correlations between the generative and estimated parameters were significant in all cases (alpha: *r* = 0.59, *p* < 0.001; v0_not_smoke: *r* = 0.59, *p* < 0.001; v0_smoke: *r* = 0.68, *p* < 0.001; beta: *r* = 0.51, *p* < 0.001). Correlations remained elevated (*r* > 0.50) when restricted to participants who completed relatively lower numbers of trials (<20 trials).

#### 2.4.3. Multi-Level Mediation and Regression Analyses

To determine trajectories across time for expected values and contentment scores for smoking and not smoking, number of craving tool uses was entered as a first-level predictor of reward values for each action. Separate models were conducted on each outcome variable. To determine significant differences in slopes between types of trial (smoking vs. not smoking), we also computed the Number of Craving Tool Uses X Trial Type (smoking or not smoking) interaction on each outcome variable (either expected reward values or contentment scores) separately.

Multi-level mediation analyses were conducted using SPSS (using MLMED computational macro) statistical analysis software package (SPSS, Inc., Version 25.0, IBM Corp., Armonk, NY, USA) to examine whether changes in smoking across trials were mediated by changes in expected reward values across time. To do this, the number of craving tool uses was entered as a first-level predictor, smoking behavior as the outcome variable, and expected reward values as the mediator. To explore the moderating role of number of mindfulness training modules completed by participants on changes in either expected reward or smoking behavior, we examined the Modules X Number of Craving Tool Uses interaction on each outcome variable (expected reward values or smoking behavior) separately. Individual subject was included as a 2nd-level predictor in all models (multi-level regression and mediation).

Finally, to evaluate the predictive relationship between expected values of smoking, and present-moment reward values at the next encounter with the behavior, multi-level regression models were also conducted, using expected reward values as first-level predictors of present-moment reward values.

## 3. Results

### 3.1. Trajectories across Craving Tool Uses for Expected Reward Value of Smoking and Not Smoking

**Figure 2:**
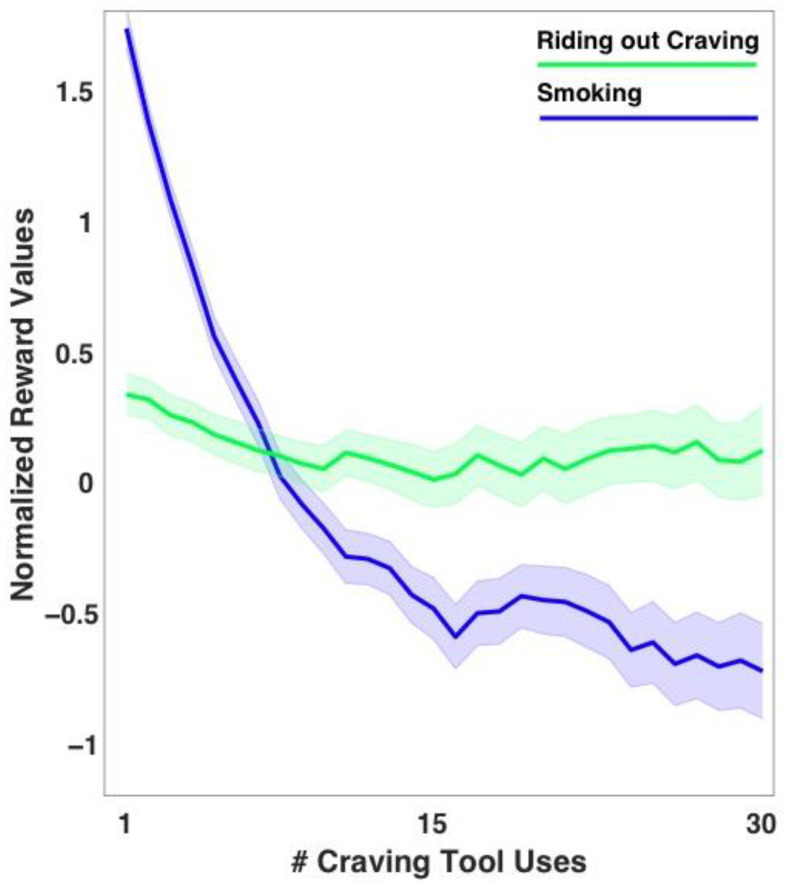
Normalized expected reward values by craving tool use (averaged across participants) for smoking and not smoking by “riding out” their craving with a brief informal mindfulness practice (RAIN exercise). For display purposes, expected values were generated using group-averaged initial values for each action as V0.

Multi-level regression analyses revealed the following slope trajectories for smoking and non-smoking trials, respectively: *B* = −0.018, *SE* = 0.003, *t* = −6.59, *p* < 0.001; *B* = −0.006, *SE* = 0.002, *t* = −2.72, *p* = 0.008. Expected values for each action (averaged across participants) across number of craving tool uses are shown in Figure 2.

The significant Number of Craving Tool Uses X Trial Type interaction (*B* = −0.012, *SE* = 0.003, *t* = −4.54, *p* < 0.001) revealed that the slope for smoking expected reward value was significantly more negative than that of the slope for expected value of not smoking, indicating that while expectations of reward might have generally decreased across tool uses, a greater decrease in expected reward was observed specifically for expected values of smoking across uses of the mindful smoking craving tool.

Relatedly, slope trajectories of contentment ratings across craving tool uses revealed a trend for a significantly negative slope trajectory of contentment from smoking (*B* = −0.003, *SE* = 0.002, *t* = −1.86, *p* = 0.072), no significant change in contentment from not smoking across trials (*B* = 0.001, *SE* = 0.002, *t* = 0.73, *p* = 0.472), and no significant difference between slopes (Trial type X Number of Craving tool use interaction: *B* = −0.002, *SE* = 0.002, *t* = −1.33, *p* = 0.192).

### 3.2. Expected Reward Value Mediates the Relationship between Craving Tool Uses and Smoking Behavior

**Figure 3:**
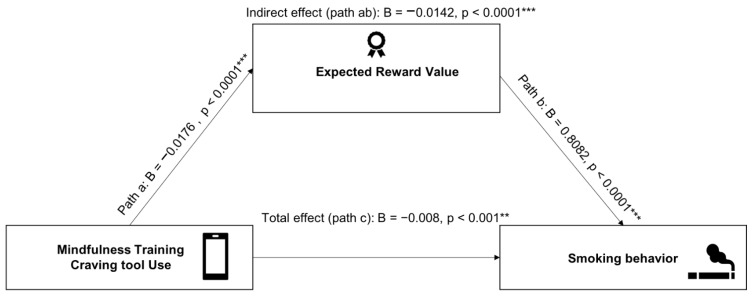
Mediation diagram for the impact of time (predictor) on smoking behavior using expected reward values as a mediator. ** *p* < 0.001, *** *p* < 0.0001.

Our multi-level mediation analyses revealed a significant negative effect of number of craving tool uses on expected reward values (path a: *B* = −0.0176, *SE* = 0.003, *t* = −6.55, *p* < 0.0001). Second, we observed a significant positive effect of expected reward values on smoking (path b: *B* = 0.8082, *SE* = 0.084, *t* = 9.58, *p* < 0.0001). Third, results revealed a significant total effect of number of craving tool uses on smoking, without accounting for a mediator effect (path c: *B* = −0.008, *SE* = 0.0017, *t* = −4.74, *p* < 0.001). Fourth, we observed a significant mediating effect of expected reward values on the relationship between number of craving tool uses and smoking (path ab: *B* = −0.0142, *SE* = 0.003, *Z* = −5.39, *p* < 0.0001). Finally, the direct effect of number of craving tool uses on smoking (after controlling for the mediator) was not significant (path c’: *B* = −0.0005, *SE* = 0.0006, *t* = −0.988, *p* = 0.3568). These results are shown in Figure 3. No significant Module x Number of craving tool use interaction on either expected reward values of smoking or smoking behavior were found (all *p*’s > 0.133).

### 3.3. Expected Reward Values Predict Present-Moment Reward Values

Expected reward values significantly positively predicted present-moment reward values in our multi-level regression model (*B* = 0.075, *SE* = 0.029, *t* = 2.62, *p* = 0.010). In other words, low expected reward values predicted low present-moment reward values, and vice versa; elevated expected reward values predicted elevated present-moment reward values.

## 4. Discussion

The present findings indicate that smoking behavior and expected reward values estimated from a reinforcement learning model exhibit negative slope trajectories across craving tool uses of an app-based mindfulness training program. Reward obtained from smoking (contentment ratings) also showed a (trend for) significantly negative slope trajectory across uses of the mindful smoking craving tool, and a significant correlation between the present moment and expected reward values across craving tool uses was found. Additionally, our results show that expected reward values in the RL model significantly mediate the relationship between the number of uses of the craving tool and decreasing slopes in smoking behavior. This supports the hypothesis that mindfulness training may engage the intended mechanistic target, and that this may account for decreases in smoking across uses of the craving tool.

The fact that mindfulness training predicted decreasing slope trajectories in smoking behavior across craving tool uses is in line with our a priori hypotheses and is consistent with results obtained from previous studies [31]. For example, the previous study by Elwafi and colleagues [31] showed that participants who underwent MT exhibited reduced smoking, as well as a decoupling between craving and smoking after 4 weeks. In addition, our results are also in line with evidence of smoking reduction [13,16,18] cessation and relapse prevention [13], as well as smoking abstinence rates [14] from mindfulness-based approaches. Although no causal inference can be made with respect to mindfulness training and our dependent variable due to the single-arm nature of our design, our results may suggest a mechanistic explanation underlying the impacts of MT on smoking behavior for future randomized-controlled trials (RCTs) to investigate.

We expanded on a previous study by Elwafi and colleagues [31] in two ways. First, we implemented a craving tool which delivers MT “on-demand” as cue-induced craving experiences arise “in vivo” in participants’ real-life settings. Here, we showed that a reinforcement learning model was a good fit for behavior, and that repeated mindful attention towards the experience and outcome of smoking was associated with a decreased trajectory of the reward value of smoking in that model. Our results suggest this may have operated by decreasing the expected reward value of smoking across uses of the craving tool. Moreover, we showed that decreased expected reward value predicted reduced present-moment reward value on the subsequent use of the craving tool. This could reflect that the use of the tool causes a break in the cycle between outcome, reinforcement of craving-inducing cues, and automated ensuing behavior [8]. In other words, reducing the reward value of smoking through mindfulness (i.e., by attending to and learning from its experiential consequences) may reduce the strength of cue-induced cravings the next time they become triggered. This suggests a successful disruption of the cycle of reinforcement in smoking addiction. Nonetheless, these hypotheses are tentative at best due to the non-experimental nature of our design, and they remain to be tested in future RCTs. In addition, the lack of effect of mindfulness training modules completed by participants on changes in expected reward values or smoking behavior indicate that results did not differ depending on levels of exposure to mindfulness concepts/techniques depicted in the program’s capsules. It is possible that participants may have applied strategies to their cravings other than mindfulness when using the app. Thus, future studies directly comparing the use of our craving tool with other craving modulation techniques such as distraction or suppression are needed to rule out other potential mechanistic confounds.

Our hypothesis that reward values would mediate the relationship between the use of the tool and smoking behavior was also confirmed. This result is in line with previous evidence that mindfulness-based approaches that target reward processes influence smoking behavior [17,18] or that reductions in expectations of reward from other unhealthy habits and behaviors, (such as maladaptive eating) are associated with a decrease in such behaviors [22]. This result is also consistent with the notion that the underlying mechanisms for which mindfulness influences behavior is by targeting reinforcement learning processes [8,17,19].

The observed mediation of the relationship between the use of the tool and smoking by expected reward values supports the notion that, in accordance with the Science of Behavior Change approach [32], mindfulness training, and the craving tool in particular, engages the hypothesized mechanistic target—reward value reduction. In addition, target engagement accounts for the desired behavioral change (smoking reduction) [32]. Nonetheless, whether MT engages the mechanistic target remains to be confirmed in experimental RCT designs from which causal inferences can be drawn.

However, future studies should also examine other plausible mechanisms using RCT designs. As such, it is possible that other mechanisms can be engaged as well. For instance, it is possible that MT leads to decreased reactivity to smoking cues [33], because MT fosters being present with unpleasant states as opposed to avoiding or changing them. As was shown by Janes and colleagues [33], reduced brain reactivity to smoking cues in a region involved in getting caught up in experience (the posterior cingulate cortex) was associated with cigarette consumption reduction in a group assigned to MT over 8 months. It is possible that the craving tool also engaged this mechanism, which will be important to test in future research. In general, this work may indicate that the very act of noticing when smoking cues/cravings arise in real-time and performing the exercise, and “pausing” before automatically carrying out a habituated behavior, could attenuate reactivity to cues and the experience of becoming “caught-up” in a craving. These interpretations, however, remain tentative until replicated in experimental RCT studies using a control group.

Finally, our finding that MT reduces health-detrimental habitual behavior by targeting reward value reduction parallels a recent single-arm study on maladaptive eating [22]. In that study, a similar craving tool was used to examine trajectories of the reward value of over-eating and/or eating unhealthy foods [22]. This study also showed that the use of the craving tool was associated with decreased reward value across tool uses, and that this change in reward value predicted eating behavior change. The fact that this result was also observed here suggests that this mechanism of change in MT may translate to multiple health-detrimental clinical outcomes that are formed and maintained through similar processes (reinforcement learning). Future RCT studies are needed to confirm the translation of MT from smoking to unhealthy eating behaviors and examine whether this mechanism of change applies to broader ranges of clinical conditions that involve reinforcement learning (e.g., substance use, pathological gambling).

It is important to highlight that the present study is not without limitations. The single-arm and correlational nature of this study, as well as the lack of a control group, both prevent us from ruling out unspecific factors (e.g., passage of time, sense of support from a psychological smoking cessation aid, etc.) as well as drawing causal inferences on the findings presented here, and therefore warrant replication in future clinical trials. Future studies should also examine the relationship between reward value changes from mindfulness training and variables independent of, or that were acquired outside, the mindfulness training exercise (e.g., CO levels before and after training period). Finally, future studies should directly compare the use of MT and this particular craving tool to other available smoking cessation treatments to compare the sustainability of reduction rates over time.

## 5. Conclusions

In conclusion, the results of this study indicate decreasing smoking behavior across number of mindful craving tool uses, and that this may be explained by a reduction in the expected reward value of smoking. The mediation of smoking behavior slope trajectories across craving tool uses by reinforcement learning processes may be attributable to allowing for mindful attention to, and learning from, the unpleasant aspects of smoking and its consequences.

These findings provide preliminary evidence for examining reinforcement learning processes as mechanisms of change underlying mindfulness training to influence smoking behavior in RCT studies.

## Data Availability

Data included in the present article will not be made publicly available due to resource sharing specifications included in the NIMH/NIH grant supporting this project. Brown University will adhere to the NIH Grants Policy on Sharing of Unique Research Resources including the “Sharing of Biomedical Research Resources: Principles and Guidelines for Recipients of NIH Grants and Contracts” issued in December 1999, and the Data Sharing Policy (Section 8.2.3.1) from the October 2019 revision. Specifically, material transfers would be made with no more restrictive terms than in the Simple Letter Agreement or the UBMTA and without reach through requirements. Nonetheless, data access requests from academic investigators can be directed to the corresponding author.
